# Mass spectrometry imaging identifies palmitoylcarnitine as an immunological mediator during *Salmonella* Typhimurium infection

**DOI:** 10.1038/s41598-017-03100-5

**Published:** 2017-06-05

**Authors:** Heather E. Hulme, Lynsey M. Meikle, Hannah Wessel, Nicole Strittmatter, John Swales, Carolyn Thomson, Anna Nilsson, Robert J. B. Nibbs, Simon Milling, Per E. Andren, C. Logan Mackay, Alex Dexter, Josephine Bunch, Richard J. A. Goodwin, Richard Burchmore, Daniel M. Wall

**Affiliations:** 1 Institute of Infection, Immunity and Inflammation, College of Medical, Veterinary and Life Sciences, University of Glasgow, Glasgow, G12 8QQ United Kingdom; 2 AstraZeneca, Milton Science Park, Cambridge, CB4 0WG United Kingdom; 3 Biomolecular Imaging and Proteomics, Department of Pharmaceutical Biosciences, Uppsala University, Box 591 BMC, Uppsala, 751 24 Sweden; 4 School of Chemistry, University of Edinburgh, Edinburgh, EH9 3FJ United Kingdom; 5 National Physical Laboratory, Teddington, Middlesex TW11 0LW United Kingdom

## Abstract

*Salmonella* Typhimurium causes a self-limiting gastroenteritis that may lead to systemic disease. Bacteria invade the small intestine, crossing the intestinal epithelium from where they are transported to the mesenteric lymph nodes (MLNs) within migrating immune cells. MLNs are an important site at which the innate and adaptive immune responses converge but their architecture and function is severely disrupted during *S*. Typhimurium infection. To further understand host-pathogen interactions at this site, we used mass spectrometry imaging (MSI) to analyse MLN tissue from a murine model of *S*. Typhimurium infection. A molecule, identified as palmitoylcarnitine (PalC), was of particular interest due to its high abundance at loci of *S*. Typhimurium infection and MLN disruption. High levels of PalC localised to sites within the MLNs where B and T cells were absent and where the perimeter of CD169^+^ sub capsular sinus macrophages was disrupted. MLN cells cultured *ex vivo* and treated with PalC had reduced CD4^+^CD25^+^ T cells and an increased number of B220^+^CD19^+^ B cells. The reduction in CD4^+^CD25^+^ T cells was likely due to apoptosis driven by increased caspase-3/7 activity. These data indicate that PalC significantly alters the host response in the MLNs, acting as a decisive factor in infection outcome.

## Introduction

*Salmonella enterica* serovar Typhimurium (*S*. Typhimurium) causes a self-limiting gastroenteritis in humans. Transmission is through the faecal-oral route by ingestion of contaminated food or water. In the small intestine, *Salmonella* traverse the epithelial layer primarily at M cells which overlay the Peyer’s patches^1^. Although in most humans S. Typhimurium infection remains localised to the gut, in some cases of human infection *S*. Typhimurium migrates to systemic sites and is linked to clinical complications, particularly in immunocompromised, young or elderly people^2^. In a murine model of infection, the mice are more susceptible to systemic disease, which occurs after dissemination of bacteria to the lymph nodes, either after phagocytosis by migrating dendritic cells (DCs) or by swimming freely in the lymph itself^3^.

The mesenteric lymph nodes (MLN) drain the intestine and these organised lymphoid structures are crucial to the host response to intestinal pathogens. Their removal prior to *S*. Typhimurium infection causes greater systemic dissemination of bacteria and increased mortality^3^. Upon entering the MLNs, non-phagocytosed *S*. Typhimurium can be taken up by CD169^+^ subcapsular sinus (SCS) macrophages, while DCs containing bacteria penetrate into the parenchyma of the MLNs to present antigen to T helper (T_h_) cells within interfollicular regions^4^. B cells reside in follicles adjacent to the SCS region where, upon acquisition of antigen from macrophages, they migrate to interfollicular regions and present antigen to previously activated T_h_ cells, initiating the germinal centre response^4, 5^. This organised sequence of events is reliant on MLN architecture in order to correctly capture, transfer and present antigen^6^. *S*. Typhimurium infection has been shown to alter both the number and location of T and B cells in the MLNs whilst also affecting positioning of DCs^6^. Additionally lipopolysaccharide (LPS) treatment or infection disrupts the organization of CD169^+^ macrophages and this SCS disruption leads to impaired antigen presentation^7^.

T cells, and CD4^+^ T cells in particular, play a critical role in combatting *Salmonella* infection. Mice in which T cells have been ablated or are defective, increasingly succumb to infection even with attenuated strains^8–10^. Targeting of CD4^+^ T cells is therefore an important virulence strategy for *Salmonella*, and selective CD4^+^ T cell killing during infection contributes to both immune evasion and persistence^11, 12^. The selective killing of CD4^+^CD25^+^ regulatory T cells during infection also contributes to the tumour suppression effects of S. Typhimurium^13, 14^.

Whilst *S*. Typhimurium infection disrupts MLN function and subsequent induction of the adaptive immune response, little is known about the mechanism(s) or host-pathogen interactions involved. Therefore, discovery of host molecules that are differentially regulated during infection has the potential to offer new insights into these interactions and can aid in the development of novel diagnostics or targeted drugs, or may simply act as diagnostic or prognostic indicators for infection or its resolution^15–17^. To determine the identity, abundance and distribution of the host molecules that are differentially regulated during infection requires new bioanalytical approaches to be used. Mass spectrometry imaging (MSI) has previously been successfully used to image host response markers and bacterial components in infected samples^15, 18–24^. MSI is capable of detecting the distribution of proteins, lipids and metabolites across a tissue section. Crucially, MSI is not dependent on tagging the molecule of interest. Collection of full mass spectra at each sampling position means prior knowledge of the target molecules that may be involved in a process under investigation is not needed. Untargeted data collection and interrogation allows the discovery of novel molecules involved in the infectious process^25^. To date, matrix-assisted laser desorption/ionisation (MALDI) MSI is the most commonly used MS molecular imaging method as it has advantages in analysis speed, high spatial resolution and can detect a wide range of molecules that can be simultaneously imaged. MALDI imaging involves thaw-mounting thin tissue sections onto slides, which are then coated in an energy-absorbing matrix to assist in ionisation of molecules within the sample. Analytes are ionised from discrete spots across the tissue section by laser ablation and mass spectra are collected from each position. Each spectrum collected acts as a single ‘pixel’ so that an image of the distribution and relative abundance of each distinct molecule in the spectrum can be generated. Utility of MSI has been expanded by application of a range of alternative sample ionisation techniques other than MALDI, such as desorption electrospray ionisation (DESI)^26^, which offers alternative sensitivities for the detectable molecular targets.

Here, for the first time, we applied MSI to a *S*. Typhimurium murine model of infection in order to discover novel molecules mediating host-pathogen interactions. A number of candidate molecules were detected to have significantly changed in both abundance and localisation. Of these, palmitoylcarnitine (PalC), a long chain fatty acid ester of carnitine, was selected for further molecular and phenotypic characterisation and was found to play a significant role in modulating the host response in the MLNs.

## Results

### Immunohistochemistry (IHC) and MALDI - MSI to discover molecules differentially regulated during S. Typhimurium infection

**Figure 1:**
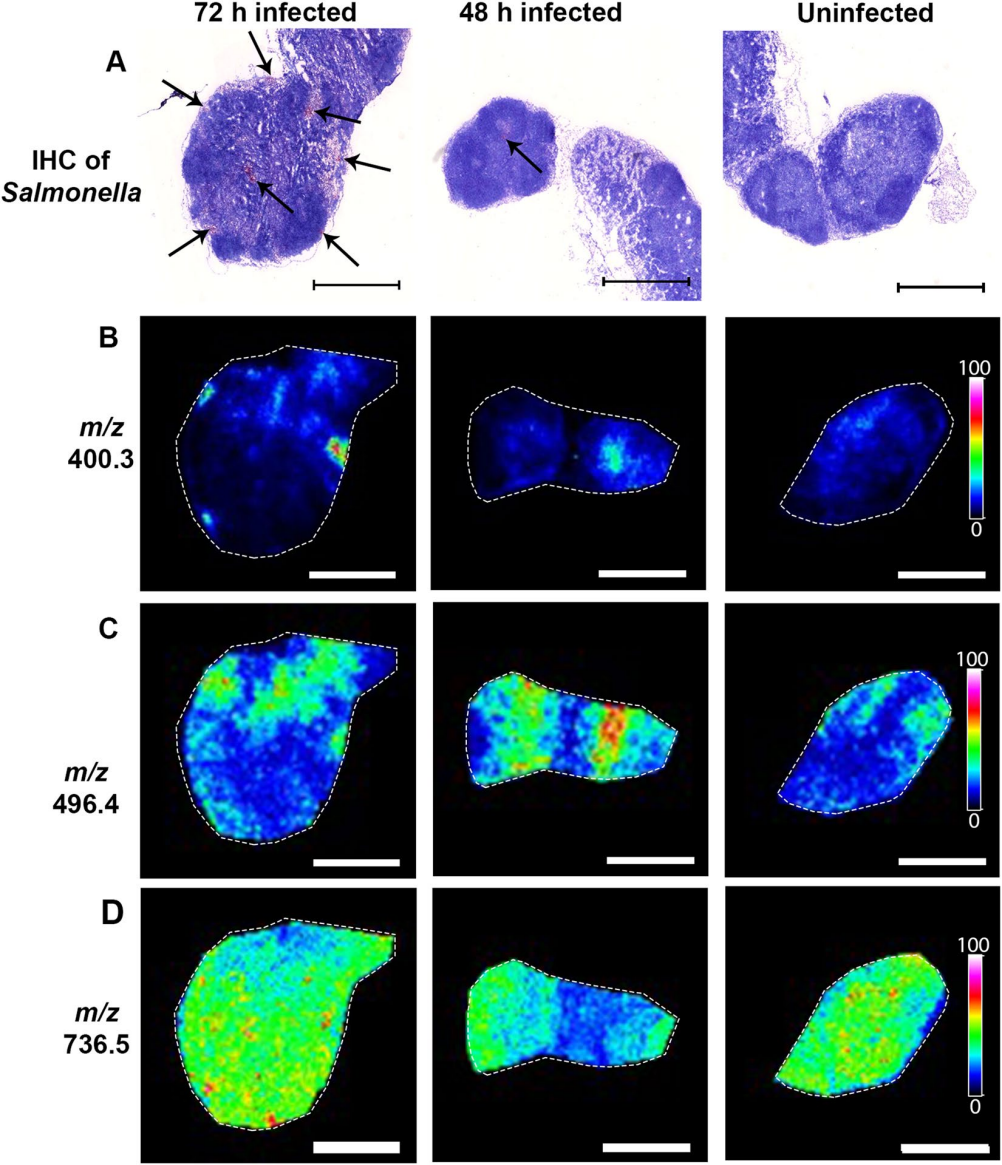
High levels of a biomarker at *m/z* 400.3 localises to areas of *S*. Typhimurium infection and to hypocellular areas of tissue disruption caused by the bacteria in MLNs from mice infected for 72 h. (**A**) IHC staining of *Salmonella* in red (arrows), showing presence of bacteria in haematoxylin and eosin stained MLNs from mice infected for 72 h. No staining was found in the MLN from a 48 h infected mouse or in the uninfected control. (**B**) MALDI-TOF MSI ion intensity maps showing the localisation and relative intensity of *m/z* 400.3 (**C** and **D**). Heat maps of *m/z* 496.4 and 736.5 were used as controls, to show there were not ubiquitous changes in small molecules in areas of damaged tissue. Colour scale bar shows the colour gradient of the MSI image in relation to relative abundance of the imaged molecule, colour gradient from 0% relative abundance of molecule to 100% relative abundance. Results are from 1 of 3 biological replicates with a representative 48 h uninfected sample shown.

Mice were infected with *S*. Typhimurium as previously described^27^. MLN sections were immunohistochemically (IHC) stained with antibodies against *Salmonella* to detect the presence of *S*. Typhimurium and the MLNs from each of the three 72 hour (h) infected mice stained positive for bacterial infiltration (Fig. 1A) (For additional biological replicates see Supplementary Figs S1a and S1e). The IHC results were used to guide MALDI-MSI, allowing identification of specific regions in the MLNs where *S*. Typhimurium had infected and where disrupted, hypocellular regions were present. Analysis of the MALDI-MSI results using MSI statistical analysis software (SCiLS lab, Bruker) indicated that 15 analytes, out of the total of 848 masses detected in the MLNs, were found to be statistically discriminative of tissue disrupted, hypocellular regions where bacteria were also present. These were found in all three biological replicates when compared to an area of the same node that was not hypocellular and where no bacteria were present (see Supplementary Table 1). One of these analytes, a molecule at a mass to charge ratio (*m/z)* of 400.3, was selected for further investigation (Fig. 1B) (For additional biological replicates see Supplementary Fig. S1). The molecule at *m/z* 400.3 was also detected at high abundance in the Peyer’s patches from 72 h infected mice that stained positive for bacterial presence, but was detected to a lesser extent in a Peyer’s patch from a 72 h infected mice where no bacteria were present (see Supplementary Fig. S2). As the Peyer’s patches were not consistently infected with *S*. Typhimurium, we concentrated our subsequent analysis on the MLNs. Although the molecule at *m/z* 400.3 was present at highest abundance in areas where there was both tissue disruption and *S*. Typhimurium present in the MLNs, it was also present in the 48 h infected and uninfected samples at lower levels. Co-localisation analysis was then carried out on *m/z* 400.3 using SpectralAnalysis software^28^, and the Matlab image processing toolbox (version R2014a, The Math-Works, Inc, Natick, MA, USA). This showed that > 70% of the signal observed for *m/z* 400.3 was localised within regions of lower cell density in the MLNs (Supplementary Fig. S3). Similarly, through principal component analysis segmentation of the MSI results, high co-localisation was observed between the low cell density regions of the MLN and the positive components of principal component 11, which m/z 400.3 has a significant contribution (PC loading > 0.1) (Supplementary Fig. S4).

### The biomarker at m/z 400.3 is palmitoylcarnitine

**Figure 2:**
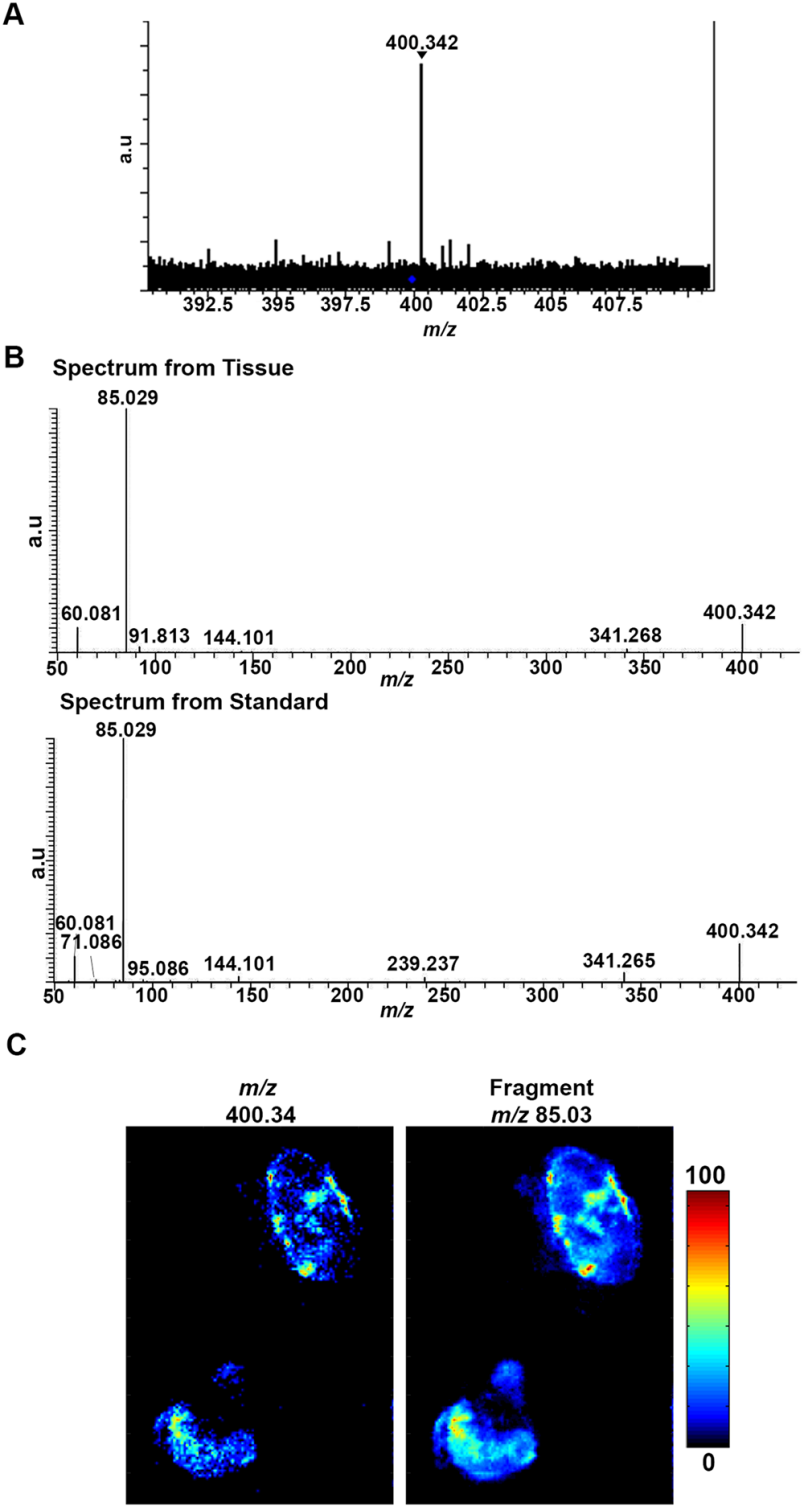
The biomarker at *m/z* 400.3 was fully identified as palmitoylcarnitine (PalC). (**A**) Mass spectrum from MALDI-FTICR-MS showing the accurate mass of the biomarker at 400.3 (400.342). (**B**) A mass spectrum from the on tissue fragmentation of the molecule at *m/z* 400.3 and from the fragmentation of the standard by DESI-Orbitrap-MSI showing the similar fragmentation pattern. (**C**) Heat map images from DESI-orbitrap-MSI validating identification by showing localisation of parent ion (*m/z* 400.34) to the same area as the main fragment from PalC (*m/z* 85.03). Colour scale bar shows the colour gradient of the MSI image in relation to relative abundance of the imaged molecule, colour gradient from 0% relative abundance of molecule to 100% relative abundance.

Due to the lower mass resolving power of the MALDI-TOF mass spectrometer used during the preliminary screening phase to detect changes in relative abundance, further mass spectrometry techniques were used to aid the definitive identification of the molecule at *m/z* 400.3. A MALDI Fourier Transform Ion Cyclotron Resonance (FTICR) mass spectrometer was used to obtain spectra with greater mass accuracy and with higher mass resolving power. This resulted in the determination of the peak at *m/z* 400.342 (Fig. 2A) to be putatively identified as palmitoylcarnitine (PalC) using the online Human Metabolome Database (http://www.hmdb.ca/). PalC has previously been implicated in bacterial infection, the proinflammatory immune response and the induction of apoptosis^29–31^. DESI-orbitrap-MSI was then used to confirm the identity of PalC (*m/z* 400.342) (Fig. 2A). This technique generates a unique molecular fragmentation fingerprint of a molecule in a tissue that can be directly compared to that of a standard. This fragmentation pattern of a PalC standard was identical to the ion fragmentation of *m/z* 400.342 seen in infected tissue (Fig. 2B). Additionally, an ion abundance heat map of the main fragment of PalC (*m/z* 85.0) indicated that it had a similar localisation to *m/z* 400.34 (Fig. 2C).

### The Effect of PalC on Bacterial Survival, Virulence and Motility

**Figure 3:**
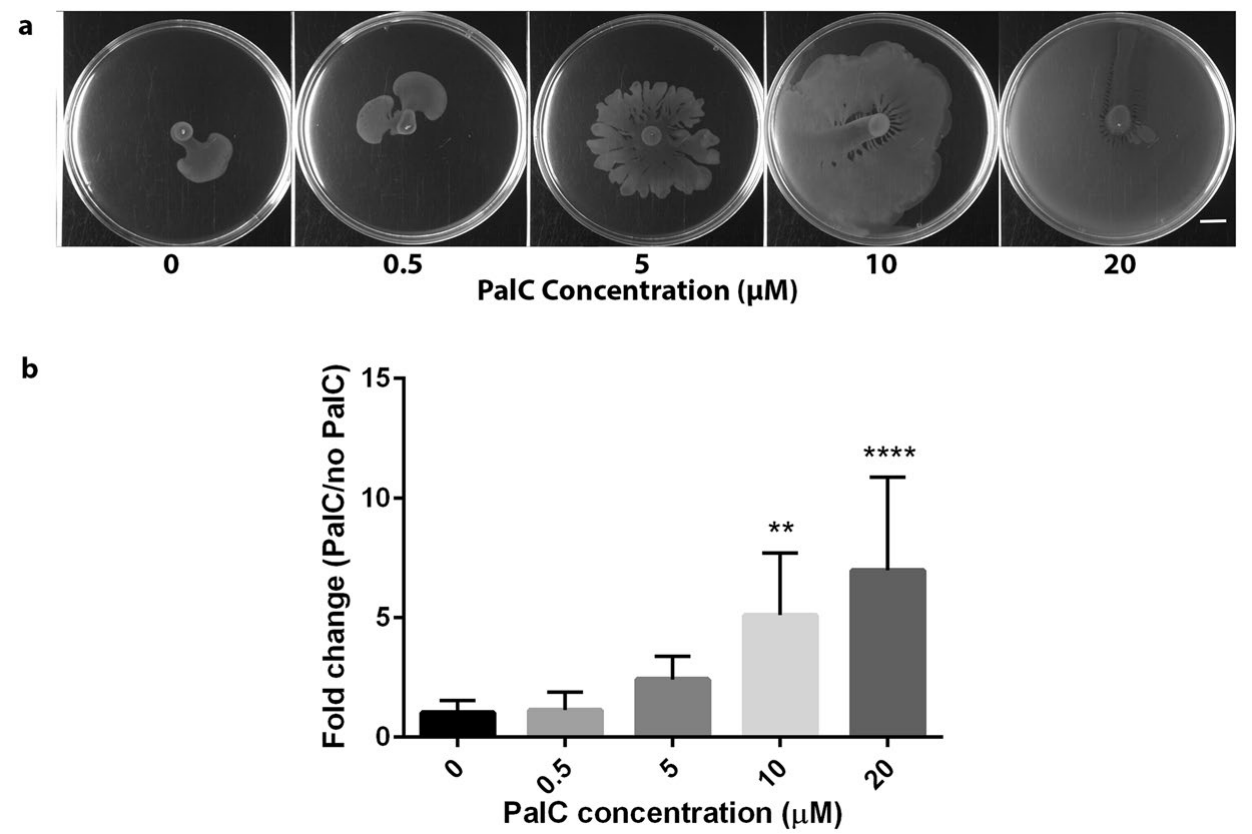
PalC increases swarming motility of *S*. Typhimurium. (**a**) *S*. Typhimurium motility was assayed on swarming plates with increasing concentrations of PalC. The images are representative of one of three technical replicates from one of three biological replicates. (**b**) Fold change in percentage of agar plate covered by swarming area, comparing PalC added at various concentrations to an untreated PalC negative control. Bars: SD (n = 9, three separate experiments, with three technical replicates per experiment); two asterisks (**) indicates significant difference compared to untreated control, P ≤ 0.01; four asterisks (****) indicates significant difference compared to untreated control, P ≤ 0.0001.

Previous studies have found PalC affects biofilm formation and bacterial motility^32, 33^. The *S*. Typhimurium wild type strain (SL1344) used here for *in vivo* infection does not form biofilms and therefore biofilm formation was not investigated in relation to SL1344 infection^34^. However, any potential anti-bacterial effect of PalC was investigated through analysis of bacterial survival and virulence in the presence of this molecule. *S*. Typhimurium (SL1344) was seen to have no growth defect due to increasing PalC concentrations in culture media (Supplementary Fig. S5a). Similarly, no PalC-dependent effect on secretion of a virulence factor, SipA, during anaerobic growth of *S*. Typhimurium was observed (Supplementary Fig. S5b). We tested the effect of PalC on bacterial twitching, swarming and swimming motility (Supplementary Fig. S5c,d). Whilst no effects were seen in twitching and swimming abilities of the strain, a significant increase in swarming was observed in *S*. Typhimurium grown on agar containing concentrations of 5 μM of PalC (P ≤ 0.05), 10 μM of PalC (P ≤ 0.01) and 20 μM of PalC (P ≤ 0.01) (Fig. 3). PalC has known surfactant properties and many bacteria produce and utilise biosurfactants during swarming. Therefore, the increased swarming observed here is most likely due to reduced surface tension on the agar caused by the surfactant properties^35, 36^. To confirm that no increase in bacterial driven motility was occurring we examined expression of the flagellar protein FliC using a *fliC-*GFP reporter system^37^. This showed that *fliC* expression was not affected by PalC (see Supplementary Fig. S5e). In conclusion, there appeared to be no direct effect of PalC on the virulence of *S*. Typhimurium although there may be an indirect effect on motility due to the surfactant properties of PalC.

### PalC localises to areas of disrupted immune cell localisation in infected MLNs

**Figure 4:**
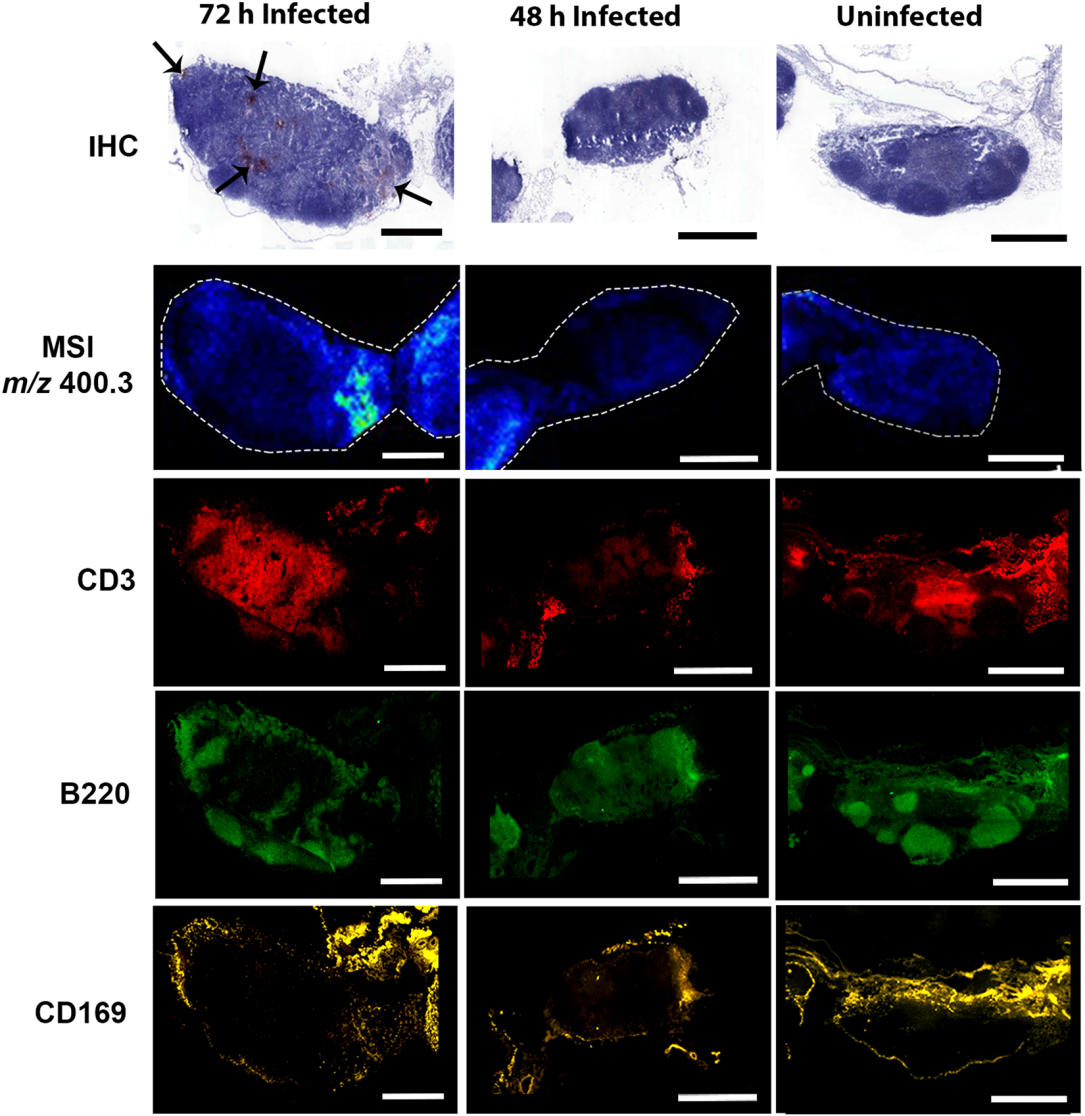
*S*. Typhimurium infection of MLNs causes disruption of B cell zones, T cells and CD169^+^ SCS macrophages, and high levels of PalC localises to these disrupted areas. IHC staining of *Salmonella* and fluorescent staining of B cells (B220^+^, green), SCS macrophages (CD169^+^, yellow) and T cells (CD3^+^, red) of MLNs from 72 h infected, 48 h infected and uninfected mice. Where *Salmonella* are present in MLNs of 72 h infected mice (red and black arrows) and where there are hypocellular areas in the IHC staining, there is disrupted architecture of B cell follicles and infiltration of SCS macrophages. The co-localisation of PalC (MSI of PalC) to the areas of disrupted B cells (B220^+^, green) and SCS macrophages (CD169^+^, yellow). There was also no T cells (CD3^+^ and red) present where PalC was localised. Scale bars, 1 mm.

Tissue sections, consecutive to those immunostained to highlight areas of *S*. Typhimurium infiltration or imaged by MALDI-MSI, were fluorescently immunostained to localise immune cells (Fig. 4). In the nodes from the 72 h infected mice, and in nodes from 48 h infected mice where bacteria were present, the architecture of B cell follicles (B220^+^) was disrupted with fewer defined B cell follicles than the MLNs from uninfected mice (Fig. 4). SCS CD169^+^ macrophage localisation was also altered in infected MLNs, with a less distinct perimeter of macrophages situated more towards the central region of the MLN rather than the periphery. The potential role of PalC in inducing these changes was examined by comparing fluorescently stained sections from the 72 h infected mice to the MALDI-MSI ion intensity map of PalC (Fig. 4). PalC localised to these areas around the perimeter of the MLN where the usually defined perimeter of CD169^+^ macrophages was disrupted in the 72 h infected node and the macrophages were migrated more centrally within the MLN. PalC also localised to areas within the MLNs where B and T cells were noted to be absent.

### PalC reduces CD4^+^CD25^+^ T cells within MLNs *ex vivo*

**Figure 5:**
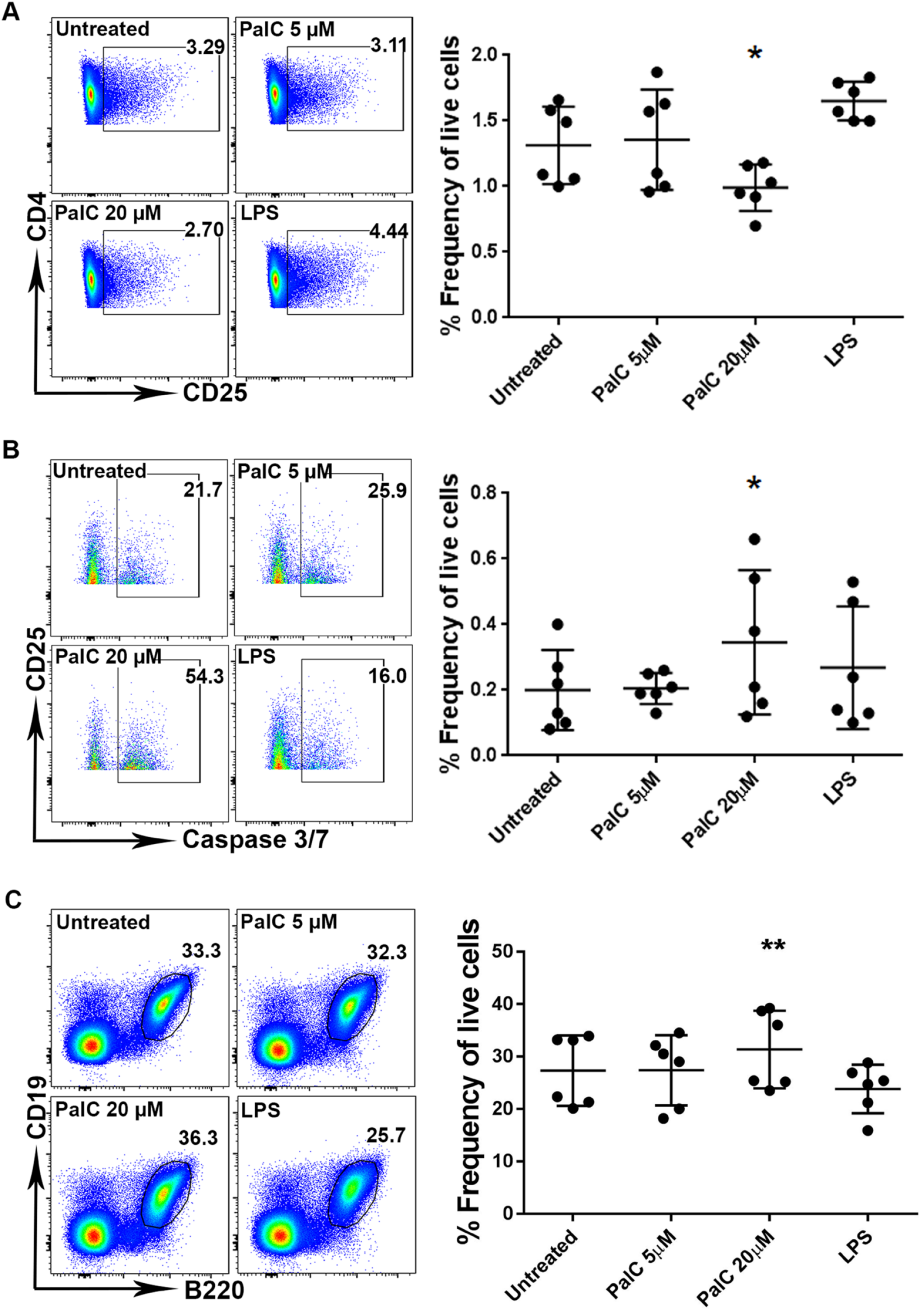
PalC causes a decrease in cell number of CD4^+^CD25^+^ T cells, an increased level of caspase 3/7 activity in CD4^+^CD25^+^ T cells and an increased number of B220^+^CD19^+^ B cells in *ex vivo* cultured MLN cells. (**A**) FACS plots of the levels of CD4^+^CD25^+^ T cells after treatment with PalC, LPS or untreated. Gating: single cells > live cells > CD45^+^ cells > CD3^+^ cells > CD4^+^ cells > CD25^+^ cells. Graph shows CD4^+^CD25^+^ T cell levels as a percentage of live cells after treatment with PalC, LPS or untreated. (**B**) FACS plots showing levels of caspase 3/7 activation after treatment with PalC, LPS or untreated. Gating: single cells > live cells > CD45^+^ cells > CD3^+^ cells > CD4^+^ cells > CD25^+^ cells > caspase-3 and -7^+^ cells. Graph shows caspase 3/7 activity levels in CD4^+^CD25^+^ T cells as a percentage of live cells after treatment with PalC, LPS or untreated. (**C**) FACS plots showing levels of B220^+^CD19^+^ B cells after treatment with PalC, LPS or untreated. Gating: single cells > Live cells > CD45^+^ cells > B220^+^CD19^+^ cells. Graph shows B220^+^CD19^+^ B cell levels as a percentage of live cells after treatment with PalC, LPS or untreated. For all FACS plots the gates show percentage of parent and the plots are a representative of 1 of 6 biological replicates. For all graphs N = 6, 6 biological replicates from 2 separate experiments. Error bars show standard deviation and one asterisk (*) = P ≤ 0.05, and two asterisks (**) = P ≤ 0.01 for multiple comparison tests.

The architecture of both B cell follicles and areas usually containing T cells were altered considerably during *S*. Typhimurium infection. Therefore, we investigated whether PalC was playing a role in directly influencing cell populations in the MLN including B and T cells, macrophages and DCs. MLNs from uninfected C57BL/6 mice were isolated and enzymatically digested before the MLN cells were cultured *ex vivo*. The effect of treatment of these cells with 20 μM and 5 μM concentrations of PalC was then examined by flow cytometry, alongside control treated and lipopolysaccharide (100 ng/ml) treated cells. No significant difference was noted in total CD3^+^ T cell frequency (Supplementary Fig. S7a), nor the CD4^+^ T cell (see Supplementary Fig. S7b), nor CD8^+^ T cell (see Supplementary Fig. S7c) frequency, under any condition tested. However, a statistically significant reduction in CD4^+^CD25^+^ T cells was noted upon treatment with PalC at 20 μM concentration (Fig. 5A). This concentration induced a 25% reduction in these cells. No statistically significant difference in MLN macrophage or DC numbers was detected under any of the conditions tested (see Supplementary Fig. S6). Additionally there was no effect of PalC on the release of inflammatory cytokines from a murine macrophage cell line (Supplementary Fig. S7).

### PalC induces caspase-3/7 mediated apoptosis in CD4^+^CD25^+^ T cells

Given the observed effect of PalC on CD4^+^CD25^+^ T cells, and the previously described ability of PalC to induce apoptosis in Jurkat cells, a T cell-like cell line, we examined PalC induction of apoptosis in T cells as well as in the specific CD4^+^CD25^+^ T cell subset^30^. Expression levels of caspase-3/7, the end point enzymatic mediators of apoptosis, were examined through flow cytometry. A significant increase in caspase-3/7 activation was seen in the CD4^+^CD25^+^ T cell population when treated with 20 μM of PalC (Fig. 5B). This indicated that during infection with *S*. Typhimurium the CD4^+^CD25^+^ T cell subset was specifically being killed in an apoptosis-dependent fashion.

### PalC increases levels of B220^+^CD19^+^ B cells within MLNs *ex vivo*

In contrast to the reduction in levels of CD4^+^CD25^+^ T cells after PalC treatment, B220^+^ CD19^+^ B cells levels were found to significantly increase by 15% upon treatment with 20 µM of PalC (Fig. 5C). The levels of caspase-3/7 activation in the B cell population did not significantly change upon PalC treatment (Supplementary Fig. S7d), so this increase is unlikely due to a reduction or inhibition of apoptosis.

## Discussion

MSI offers a unique opportunity to study host-pathogen interaction *ex vivo* because of its label-free detection and the characterisation of molecules directly from tissue sections. Furthermore, MSI offers potential for biomarker discovery^38^ as analysis can be performed untargeted, with statistical interrogation to discern molecular changes between tissue regions. MSI has grown in popularity as a tool to study host-pathogen interactions and has been used to find molecules differentially regulated due to infection^15, 18–22, 39–41^.

Here MSI was used for the first time to identify novel differentially expressed host markers of infection in an *S*. Typhimurium infection model. Several molecules were found in increased abundance in small, disrupted hypocellular areas in the MLNs from mice infected for 72 h, where they colocalized with *S*. Typhimurium. PalC, an acylcarnitine involved in the transport of fatty acids across the inner mitochondrial membrane for β-oxidation, was identified as one of these molecules. PalC was of particular interest as this increase in infected MLNs is corroborated by previous studies that found increased levels of PalC in lymph in a rat sepsis model, while higher levels of acyl carnitines are present in the plasma of patients with bacteraemia^29, 42^. However the results obtained here suggest the increased abundance and effects of PalC during *S*. Typhimurium infection may be more localised and that PalC plays a specific role in the host response to infection. Furthermore, it is likely that this marker is not particular to *S*. Typhimurium infection of the MLN, but is a more general marker for bacterial infection, and possibly inflammation, as evidenced by increased PalC levels in the plasma of Crohn’s Disease (CD) patients and the PalC/acylcarnitine transporter mutation that has been linked to CD^43, 44^.

We hypothesised that PalC may function in a number of ways during infection; either as a host anti-bacterial molecule, a local immunomodulatory molecule, or as a by-product of damage or increased energy requirement of inflammation. PalC has previously been found to have anti-bacterial properties, inhibiting biofilm formation and inducing bacterial motility^32, 33^. As the wild type strain of *S*. Typhimurium, SL1344, used here does not form biofilms this ability was not tested, but PalC effects on growth, virulence and motility were analysed^34^. A difference was found only in the swarming motility of the bacteria, which increased with increasing levels of PalC. However, as we found no effect of PalC on increased flagellar protein expression, it is likely that this increased swarming is due to the surfactant properties of PalC. Furthermore, as swarming motility is characterised by movement across a solid surface, this effect of PalC may not be physiologically relevant in the MLN environment where *S*. Typhimurium would likely be intracellular or moving between cells^45^.

The MLNs during *S*. Typhimurium infection were investigated by MSI as the MLNs form a critical filter restricting the dissemination of infiltrating pathogenic bacteria. Here decisive interactions between the host immune system and invading pathogens take place during infection and can result in either the systemic spread of bacteria or the induction of a controlled immune response and resolution of infection^3^. While the outcomes of these interactions in the context of the host-pathogen relationship have been well characterized, little is known of the molecules that mediate these outcomes. Similar to previous studies we noted that both the T and B cell zones in the MLNs were found to be disrupted during *S*. Typhimurium infection^6^. Furthermore, the perimeter of CD169^+^ SCS macrophages was disrupted as has previously been found during inflammation and *Staphylococcus aureus* infection, but to our knowledge has not previously been shown during infection with *Salmonella* ^7^. PalC localised to these areas of architectural disruption, where T and B cells were absent and around the SCS region where CD169^+^ cells were disrupted and now situated more centrally within the MLN. This architectural disruption in the MLNs at sites where PalC is present benefits invading pathogens as antigen filtration and presentation, and immune cell interactions would be impeded^6, 7^.

In order to understand the effect of PalC on various immune cells of the lymph node, *ex vivo* cultured MLN cells were treated with PalC. PalC significantly decreased CD4^+^CD25^+^ T cell numbers and this was most likely due to induction of apoptotic cell death as increased levels of the enzymatic mediators of apoptosis caspases-3 and -7 were also detected in the PalC treated population. Whilst PalC has previously been shown to increase apoptosis in a T cell-like Jurkat cell line *in vitro*, this is the first time a role for PalC in reducing T cell numbers has been described during *ex vivo* treatment, and the first description of a role for PalC in reducing T cell numbers in such a highly targeted manner during infection^30^.

The killed CD4^+^CD25^+^ T cells could be activated, proliferating or regulatory (Treg) T cells and PalC mediated killing of these cells could have either a dampening or proinflammatory effect on the immune response. *Salmonella* infection suppresses the immune system in a number of ways including through the specific killing of activated CD4^+^ T cells^11, 12^. While previous work on the killing of CD4^+^ T cells involved a persistent *Salmonella* infection model compared to the acute infection model used in the present study, the role of activated CD4^+^CD25^+^ T cells in inducing an immune response against intracellular bacteria means they represent an obvious target for *S*. Typhimurium induced killing, even at such an early stage of infection^46^. Therefore it is possible that the PalC dependent depletion of CD4^+^CD25^+^ T cells observed here could be a *S*. Typhimurium driven immune evasion strategy. Further investigation would be required to determine whether the reduced CD4^+^CD25^+^ T cell population after PalC treatment are activated T cells rather than FoxP3^+^ Treg cells, as the CD25 interleukin-2 receptor is common to both these cell types^46^. However, several previous studies have shown that greater than 80% of CD4^+^CD25^+^ cells in the MLNs are FoxP3^+^ Treg cells and therefore the majority of CD4^+^CD25^+^ cells in the present study are again likely to be Treg cells^47–49^. Treg cells supress the proinflammatory immune response and control the tight balance between pathogen killing and self-damage^50^. Mice infected with attenuated anti-tumour *S*. Typhimurium have similarly been noted to have decreased numbers of CD4^+^CD25^+^ cells. The consequent reduction in these regulatory cells in tumours was speculated to allow tumour infiltration of cytotoxic T cells, potentially contributing to the potency of *S*. Typhimurium in tumour reduction and killing^13, 14^. A similar killing of Treg cells here, by PalC during infection, cannot be discounted as a host response to bacterial infiltration of the MLNs, enhancing inflammation to aid in resolving the infection. However, there is other evidence to suggest that reduced Treg cell numbers could also be detrimental to the host response, as increased number and activity of Treg cells after feeding mice a probiotic is protective against *Salmonella* infection and the damage caused by the bacteria^51^. The cell disruption we observed in the MLN, where PalC localised, could point to excess damage caused by reduced control of the inflammatory response by Treg cells.

In contrast to the decrease in CD4^+^CD25^+^ T cells following PalC treatment, B220^+^CD19^+^ B cells were found to significantly increase after treatment. This increase is unlikely due to decreased apoptosis of these cells as there was no difference found in the levels of caspase-3/7 levels in this population. Further investigation is needed to understand whether the B cells are proliferating in the presence of PalC, however, this seems unlikely as a previous study showed B cell proliferation to be inhibited by this molecule^52^. It is possible that PalC is causing increased survival in the B cell population, which could be advantageous to *S*. Typhimurium as an immune evasion strategy. *S*. Typhimurium can infect B cells and survives by inhibiting pyroptotic cell death as a means to avoid the immune response, thus contributing to the dissemination of *Salmonella* ^53–55^.

Whilst the effects of PalC were defined, the origin of PalC is not yet known. It appeared in hypocellular regions where bacteria were not always present leading us to conclude that PalC was host derived and likely released by infected or dying cells. Previous work on changes in host gene expression after *S*. Typhimurium infection in the colon of infected mice highlighted a decreased expression of enzymes involved in fatty acid metabolism downstream of PalC which could lead to accumulation of acylcarnitines^56, 57^. It is possible that *S*. Typhimurium are having a similar effect on fatty acid metabolism on cells within the MLNs, causing accumulation of PalC in infected cells and in turn affecting surrounding uninfected immune cells.

This study clearly demonstrates the ability of MSI to find novel molecules involved in host pathogen interactions. PalC had no known direct influence on the immune response *in vivo*, despite research confirming its presence in bacterial and inflammatory diseases^29, 42, 43^. Here, for the first time through the use of MSI, we have spatially and temporally linked *S*. Typhimurium infection to a specific role for PalC in killing a T cell subset. This is the first study to use MSI to look for molecules involved in *Salmonella* infection and this technology has shown great promise in not only identifying novel compounds during infection but in helping to assign a role to PalC, a compound previously implicated in host-pathogen interactions. Further studies using this technology will aid our understanding of the host-pathogen relationship and will reveal new compounds that may be exploited to modulate the response to infection and inflammation.

## Methods

### Mouse Model of Infection

Eight to ten week old female C57BL/6 mice were orally administered 3.75 mg of streptomycin in 100 µl of sterile water 24 hours (h) prior to infection^27^. Mice were infected with 5 × 10^7^ colony-forming units (cfu) per millilitre (ml) of wild type *S*. Typhimurium (SL1344) in 100 µl of sterile phosphate buffered saline (PBS) by oral gavage or 100 µl of sterile PBS for uninfected control mice. Forty eight and 72 h post treatment mice were euthanized and tissue collected. Approval for these procedures was given prior to their initiation by an internal University of Glasgow ethics committee and all procedures were carried out in accordance with the relevant guidelines and regulations as outlined by the U.K. Home Office (PPL 70/8584).

### Tissue Processing for MSI and Histology

MLNs were removed and embedded in 2.5% medium viscosity, carboxymethyl cellulose (Sigma-Aldrich) in distilled water prior to snap freezing in a slurry of ethanol and crushed dry ice, then stored at −80 °C until processing for MSI and histological analysis. Six micrometre (μm) thick sections were cut from frozen tissue using a cryostat microtome (Thermo Scientific). Sections were cut in a specific order for MSI and histology techniques: three consecutive sections were cut for haematoxylin and eosin staining or immunohistochemistry (IHC) and mounted onto glass slides; then two adjacent sections were cut for MSI and thaw mounted onto conductive indium tin oxide (ITO) coated slides (Bruker Daltonics) for MALDI-MSI and MALDI FTICR MS, and normal non-conductive microscope slides for DESI-orbitrap-MSI. This cutting sequence was repeated until a sufficient number of slides for MSI were obtained. All slides were stored at −80 °C following sectioning.

### MALDI-TOF Mass Spectrometry Imaging

Slides for MALDI-MSI were removed from −80 °C and immediately desiccated for 30 min before matrix application. Alpha-cyano-4-hydroxycinnamic acid (CHCA, 5 mg/ml) in 50:50 acetonitrile: water and 0.2% trifluoroacetic acid was applied using an automatic matrix sprayer (TM-Sprayer, HTX Technologies) at 0.08 ml/min, 95 °C, with 6 passes. The MSI experiments were carried out using the ultrafleXtreme MALDI TOF/TOF instrument (Bruker Daltonics) equipped with a 2 kHz smartbeam laser. The raster width for imaging runs was 25–30 μm and 300 laser shots were collected at each raster position with a mass to charge ratio (*m/z*) range of approximately 100–1000. The imaging data was analysed using either FlexImaging 3.0 or 4.0 software (Bruker Daltonics) and normalised by total ion count.

### MSI Analysis to Find Discriminative Analytes of Hypocellular, Bacteria Areas

In total, 6 hypocellular regions were compared to 6 non-hypocellular regions in the 3 biological replicates of the MLNs from the 72 h infected mice. Firstly, receiver operating characteristic (ROC) analysis was performed to identify m/z values, which discriminate between the two different region types. Molecules with an area under the curve (AUC) value of >0.8 were selected for further analysis, as these would be the most highly discriminative. An Anderson-Darling test was performed to determine that these values were not normally distributed (p-value > 0.05) and therefore a non-parametric, Wilcoxon test was chosen for statistical analysis. Molecules with an AUC value of >0.8 and a *p-value* of <0.05 were deemed to be discriminative of the hypocellular, bacteria present regions^58^. Data processing used to calculate the co-localisation of low cell density regions of the MLNS with the MSI data is described in detail in the supplementary information.

### Accurate Mass Analysis of molecule of interest by MALDI FTICR Analysis

A 12 T SolariX FTICR MS (Bruker Daltonics) was used to acquire high mass accuracy spectra for the ion of interest at *m/*z 400.3, previously detected by MALDI-TOF-MSI. Slides with infected and uninfected tissue sections prepared for MSI were used and matrix applied as previously described. Areas of the tissue sections to be analysed were identified using the MSI results and an optical image of the slide taken using a flatbed scanner (Epson Perfection V370). The laser diameter was set to medium and 50 μm areas of tissue were ionised by random walk. A spectrum with a mass range of *m/z* 200–2000 was acquired for each sample position and continuous accumulation of selected ions (CASI) was used to increase the signal-to-noise of the ions in the range *m/z* 400.34 ± 5. Data was analyzed using Xcalibur 2.2 (Thermo Scientific) to gain an accurate mass of the molecule at 400.3 and the *m/z* ratio was searched on the online human metabolome database (http://www.hmdb.ca/) to gain a preliminary identity.

### Analysis using DESI-Orbitrap-MSI for validation of molecule of interest identity and localisation

DESI-MSI was performed on a Thermo Scientific Q-Exactive mass spectrometer equipped with an automated Prosolia 2D DESI source. A home-built DESI sprayer assembly was used with the spray tip positioned at 1.5 mm above the sample surface and at an angle of 75°. The distance between the sprayer to mass spectrometer inlet was 7 mm with a collection angle of 10°. The spray solvent was methanol/water (95:5 v/v), delivered at 1.5 μL/min using a Dionex Ultimate 3000 pump (Thermo Scientific) at a spray voltage of 4.5 kV. Nitrogen was used as the nebulisation gas at a pressure of 7 bars. The Q-Exactive mass spectrometer was operated in positive ion mode for all analysis using an S-Lens setting of 50 V. To acquire full mass spectra in the mass range of *m/z* 150–600 a mass resolution of 140000, AGC target of 5000000 and injection time of 200 ms was used. For acquisition of MS/MS spectra, a mass range of *m/z* 50–425 was used together with an injection time of 150 ms, AGC target of 1000000 and mass resolution of 35000. Fragmentation was performed using HCD setting of 30 and a mass isolation window of ± 0.3 Da. For distribution of the parent mass of *m/z* 400 a spatial resolution of 100 μm was used, while distribution of the fragment ion was acquired using 85 μm spatial resolution and corresponding scan speed was 130.21 μm/s and 472.22 μm/s respectively. Before analysis, samples were dried under a nitrogen stream for one minute, and full scan and fragmentation analysis was performed on adjacent tissue sections. For PalC standard analysis, PalC solubilized in DMSO was spotted on a slide and the spot was scanned to collect a spectrum. Data was converted into imzML format using imzML converter version 1.1.4.5 and data was visualised using MSIReader version 0.06^59^. All intensities shown are raw ion intensities and 1^st^ order linear interpolation was used for image generation.

### The Effect of PalC on S. Typhimurium motility

PalC, solubilised in water was added to the agar plates prior to pouring for the motility assays and water alone was added to plates as an untreated control. Twitching assays were performed by stab inoculating 1.5% LB agar plates, swarming assay plates were set up by inoculating the centre of 0.6% agar plates, supplemented with 0.5% glucose, with 6 µl of culture and swimming assays were performed by inoculating 6 µl of culture to the centre of 0.3% agar. All plates were inoculated with SL1344 from an overnight culture in LB media. The plates were incubated at 37 °C overnight for twitching plates and for 6 h and 8 h for swimming and swarming assays respectively, or until the bacteria filled the whole area of the plate. Statistical analysis was performed with Prism 6.07, Graphpad, a one-way ANOVA followed by a Dunnett’s multiple comparisons test to compare each sample to the untreated control.

### The effect of PalC on S. Typhimurium growth, FliC motility and virulence

To analyse the effect of PalC on bacterial growth, *S*. Typhimurium (SL1344) were grown in the presence of PalC at various concentrations and the CFU/ml of the bacteria was assessed over a 24 h period. For the effect of PalC on FliC regulated motility, levels of *flic* expression were assessed by transforming *Salmonella* with a pAJR153 (*fli*c:GFP) plasmid and levels of fluorescence were analysed after treatment with PalC. Finally, to study the effect of PalC on *Salmonella* virulence, the levels of type 3 secreted effector protein, SipA, after treatment of bacteria with various concentrations of PalC was analysed. For detailed methods of the effects of PalC on *Salmonella* see supplemental experimental procedures.

### Immunohistochemistry and Immunofluorescence Staining

Slides of MLN sections for immunohistochemistry (IHC) staining the for presence of *S*. Typhimurium were removed from −80 °C and dried at room temperature for 10 min. Slides were then fixed with 4% paraformaldehyde (PFA) in phosphate buffered saline (PBS) for 10 min then washed three times in PBS. The sections were blocked in 10% normal goat serum (NGS) for 1 h, and then in 0.2% Triton in PBS for 10 min and washed for 5 min in PBS. Finally, the sections were blocked with 0.3% H_2_O_2_ in PBS for 10 min then washed in PBS. The anti-*Salmonella* antibody (Abcam, ab53299) was used at a concentration of 1:150 in 10% NGS and incubated overnight at 4 °C. The slide was rinsed in PBS followed by three x 5 min washes in 0.5% PBS Tween-20. VECTOR NovaRed peroxidise substrate kit (Vector Laboratories) was used according to manufacturer’s instructions and the sections were counterstained with haematoxylin and mounted with DPX mounting media (Atom Scientific). Slides were scanned and images produced with Leica Slidepath software.

To determine cell types that localise to areas where PalC is being produced, B cells, T cells and CD169 SCS macrophages were fluorescently stained. Tissue sections were fixed in 4% PFA, washed in PBS, blocked for 45 min in 10% NGS in PBS and then blocked with Avidin/Biotin blocking kit (Vector Laboratories), according to manufacturer’s instructions. B cells were stained with a B220 antibody (Biolegend Clone RA3-6B2) at a 1:200 dilution, T cells were stained with a CD3ε biotin conjugated antibody (eBioscience Clone 145–2C11) at a 1:150 dilution and secondary streptavidin Alexa Fluor 555 conjugated antibody (ThermoFisher), 1:500, and SCS macrophages were stained with a CD169 antibody (eBioscience. Clone SER-4) at a dilution of 1:200. All antibodies were diluted in 10% foetal calf serum (FCS) in PBS. The sections were washed twice for 5 min in PBS and the slides were mounted with Vectashield Hardset with DAPI (Vector Laboratories). Fluorescent images were taken using the EVOS cell imaging system (Life Technologies).

The backgrounds were lowered and the colour levels brightened in both the IHC stained images and the fluorescent stained images using Adobe Photoshop CS6, this was done equally for all 3 conditions (untreated, 48 h treated and 72 h treated).

### Lymph Node Cell Analysis for Activation and Cell Death

LNs were collected from 6–8 week old mice and digested in 3 ml of RPMI-1640 with 0.8 mg/ml of dispase (Roche), 0.2 mg/ml of collagenase P (Roche) and 0.1 mg/ml of DNase I (Invitrogen). The LNs were digested for 20 min before breaking up by pipetting and collecting the cells which were then diluted to a concentration of 6.25 × 10^5^ cells/ml and cultured as previously described^60^. The cells were plated 2 ml/well in 6 well plates and incubated for 3 h at 37 **°**C before addition of 500 μl of the culture media containing the different treatments for final well concentration of: 5 μM PalC; 20 μM PalC; 100 ng/ml LPS as a positive control for infection and an untreated control. The final cell concentration was 5 × 10^5^ the cells were incubated at 37 **°**C and 5% CO_2_ for 16 h and the cells for each condition were pelleted by centrifuging at 400 g for 5 min. The cells were washed in PBS and stained with fixable viability dye (eBioscience), 1:1000 in PBS, for 15 min. The cells were washed with FACS buffer (PBS, 2 mM EDTA and 2% FCS) and separated into different tubes for staining with antibody panels for either T cells or B cells/monocytes. For T cell analysis, cells were stained with antibodies to CD45 (clone 30-F11, Biolegend), CD3 (clone 17A2, Biolegend), CD4 (clone GK1.5, Biolegend), CD25 (clone PC61, Biolegend), and CD8a (clone 53–6.7, Biolegend). For B cell, macrophage and DC analysis, cells were stained with antibodies for CD45 (clone 30-F11, Biolegend), B220 (clone RA3-6B2, Biolegend), CD19 (clone 6D5, Biolegend), CD11c (clone RA3-6B2, Biolegend), MHCII (I-A/I-E) (clone M5/114.15.2, Biolegend) and F4/80 (BM8, Biolegend). The cells were washed fixed with 100 μl of fixation buffer (Biolegend) for 20 min before washing and re-suspending in FACS buffer. Prior to fixation, the cells for caspase analysis were stained using the Vybrant FAM Caspase-3 and -7 assay kit (ThermoFisher Scientific), according to manufacturer’s instructions. Cells were analysed for each treatment on a BD LSR II (BD Biosciences) and data were analysed with Flowjo software (Flowjo). T cells were gated single cells > live cells > CD45^+^ > CD3^+^ > CD4^+^ (or CD8^+^) > CD25^+^ > caspase-3 and -7^+^. B cells were gated single cells > live cells > CD45^+^ > B220^+^CD19^+^ > caspase-3 and -7^+^. Statistical analysis was performed using GraphPad Prism 6.07, analysis for CD3^+^ T cells (Fig. S3A), CD8^+^ T cells (Fig. S3C), CD4^+^CD25^+^ T cells (Fig. 5A), B220^+^CD19^+^ B cells (Fig. 5C), B220^+^CD19^+^ caspase-3 and -7^+^ cells (Fig. S3D) were analysed using a one way ANOVA, followed by a Dunnett’s multiple comparisons test. CD4^+^ T cells (Fig. S3B) and CD4^+^CD25^+^ caspase-3 and -7^+^ cells (Fig. 5B) were analysed using a Friedman test followed by a Dunn’s multiple comparisons test.

## Electronic supplementary material

Supplementary Material
