# Supplementary material for: Mass spectrometry imaging identifies palmitoylcarnitine as an immunological mediator during Salmonella Typhimurium infection
Source: Sci Rep. 2017 Jun 5;7:2786. doi: 10.1038/s41598-017-03100-5 (PMC5459799; doi:10.1038/s41598-017-03100-5)
Supplement: Supplementary file 1 — Supplementary Material [file 41598_2017_3100_MOESM1_ESM.pdf]

Mass spectrometry imaging identifies palmitoylcarnitine as an immunological mediator during *Salmonella* Typhimurium infection

Heather Hulme[1], Lynsey M. Meikle[1], Hannah Wessel[1], Nicole Strittmatter[2], John Swales[2], Carolyn Thomson[1], Anna Nilsson[3], Robert J. B. Nibbs[1], Simon Milling[1], Per E. Andren[3], C. Logan Mackay[4], Alex Dexter[5], Josephine Bunch[5], Richard J.A Goodwin[2], Richard Burchmore[1,6], Daniel M. Wall[1,6,*]

[1]Institute of Infection, Immunity and Inflammation, College of Medical, Veterinary and Life Sciences, University of Glasgow, G12 8QQ, United Kingdom; [2]AstraZeneca, Milton Science Park, Cambridge, CB4 0WG, United Kingdom; [3]Department of Pharmaceutical Biosciences, Uppsala University, 751 24, Sweden; [4]School of Chemistry, University of Edinburgh, EH9 3FJ, United Kingdom; [5] National Physical Laboratory, Teddington, Middlesex TW11 0LW, United Kingdom

Supplementary Information

Materials and Methods

Localisation of m/z 400.3 molecule using SpectralAnalysis software

IHC images were processed in Matlab (version R2014a, The Math-Works, Inc., Natick, MA, USA), using the statistics and image processing toolboxes. Hypocellular regions of the IHC images were segmented using the *k*-means clustering algorithm. Following this, to remove any small (single pixel) holes, a dilation followed by erosion transformation was performed, and to remove any dispersed single pixels, an erosion followed by dilation transformation was applied. MSI data were converted to imzML using the flexImaging software (Bruker Daltonics version 4.1), and loaded into the SpectralAnalysis software package [1]. A mean spectrum from these data was generated, the baseline was removed using a top hat algorithm with an element width of 5, and the resulting spectrum was peak picked using a gradient method. The top 2,075 peaks were then used to form a datacube for further multivariate analysis. Manual registration of the MSI data to the IHC was then performed using the control point registration in the Matlab image processing toolbox (version R2014a, The Math-Works, Inc., Natick, MA, USA). Automated registration using cross correlative means could be performed, however since the goal of registration was to determine the correlations between these images, this was not considered appropriate. Following registration, the degree of co-occurrence between the hypocellularity in the IHC and *m/z* 400.3 and PC 11 in the MSI data were calculated as follows. Co-occurrence = number of pixels in the registered MSI above a given threshold that localise to the segmented hypocellularity in the IHC / total pixels in the MSI above the threshold.

The Effect of PalC on S. Typhimurium growth

One hundred microliters of an overnight culture of S. Typhimurium, SL1344, was inoculated into 4900 µl of Luria Bertani broth (LB) with PalC, to make final PalC concentrations of 0, 0.1, 1, 10 and 20 µM. Cultures were grown at 37°C, shaking at 180 rpm for 24 h and the OD_{600} of the cultures was analysed at various time points, which was converted to cfu/ml.

The Effect of PalC on flic expression in S. Typhimurium

The plasmid, pAJR153(*flic::gfp*), was kindly provided by Dr. Andrew Roe, University of Glasgow. *S*. Typhimurium SL1344 was transformed with pAJR153 (*flic::gfp*)[2] by electroporation. Expression from reporter plasmids was performed as previously described[3]. Transformants were inoculated into 5 ml of LB media and grown overnight at 37°C and shaking at 180 rpm. The bacteria were added to a 96 well plate with various concentrations of PalC. Fluorescence intensity was analysed using a FLUOstar OPTIMA, fluorescent microplate reader (BMG Labtech) with an excitation filter of 485/12 nm and emission of 520 nm. Readings were taken every 10 minutes over 11.5 h, during this time the plate was incubated at 37°C whilst shaking.

Type 3 effector secretion by S.* Typhimurium *in the presence of PalC

S. Typhimurium SL1344 cultures were grown in LB broth with shaking at 37ºC for 8 h. Cultures were back-diluted 1:10 and grown overnight in a static incubator, to induce type 3 secreted effector (T3SE) secretion, in the presence of PalC at concentrations of 0, 0.5, 5, 10 and 20 µM. OD_{600} measurements were taken to ensure no growth defect occurred due to addition of PalC and Live/Dead staining was also used to ensure no detrimental effect to the bacteria of addition of PalC at the various concentrations. Cultures were centrifuged at 4000 g for 10 min to remove bacteria and the supernatant was filtered through a 0.2 µM syringe filter to remove any remaining bacteria. Protein was precipitated using 15 % trichloroacetic

acid at 4ºC for 1 h before centrifugation at 4000 g for 30 min Precipitated protein was re-extracted using 100 % acetone before centrifugation at 14000 g for 5 min. Pellets were air dried before resuspension in purified water supplemented with Complete Mini protease inhibitor (Roche). Protein was quantified and run on an SDS-PAGE gel before staining with Simply Blue stain (ThermoFisher). Levels of secreted SipA were examined by densitometry with the mutant *S*. Typhimurium EE633 (ΔSipA) strain used as a control to allow easy identification of SipA on the Coomassie stained gel.

The effect of PalC on the release of inflammatory cytokines from macrophages *in vitro*

RAW 264.7 macrophages were grown in RPMI with 3% FCS and 1% L-glutamine and were treated with 1µg/ml lipopolysaccharide overnight, then washed with RPMI prior to PalC treatment. Various concentrations of PalC (shown in the graphs in the results section) was added to the cells for 24 hours then all the media and cells were removed from well, centrifuged and supernatant collected. Supernatants were diluted 1:10 prior to cytokine analysis with ELISAs for IL6 (IL6 MAX Standard ELISA, Biolegend), TNFα (TNFα MAX Standard ELISA, Biolegend) and IL1β (IL1β MAX Standard ELISA, Biolegend), which were used according to manufacturer's instructions. Absorbance was read at 450nm using a plate-reader.

SUPPLEMENTARY FIGURES AND TABLES

Supplementary Table S1, Ratios of molecules which are statistically discriminative of disrupted, hypocellular areas of infected mesenteric lymph nodes. Asterisks show masses which could be isotope peaks.

m/z of peak	**AUC**	***p-value***
372.3	0.966895	0.0146276
398.3	0.965484	0.0146276
400.4	0.97617	0.0146276
402.3	0.819644	0.0146276*
424.4	0.956337	0.0146276
426.3	0.983364	0.0146276*
428.4	0.934598	0.0146276*
579.5	0.831336	0.0213399
678.4	0.857912	0.0168213
694.4	0.855595	0.0168213
718.5	0.804619	0.0146276
720.5	0.828799	0.0168213*
742.4	0.834335	0.0417031
759.5	0.841449	0.0146276
780.5	0.870378	0.0213399

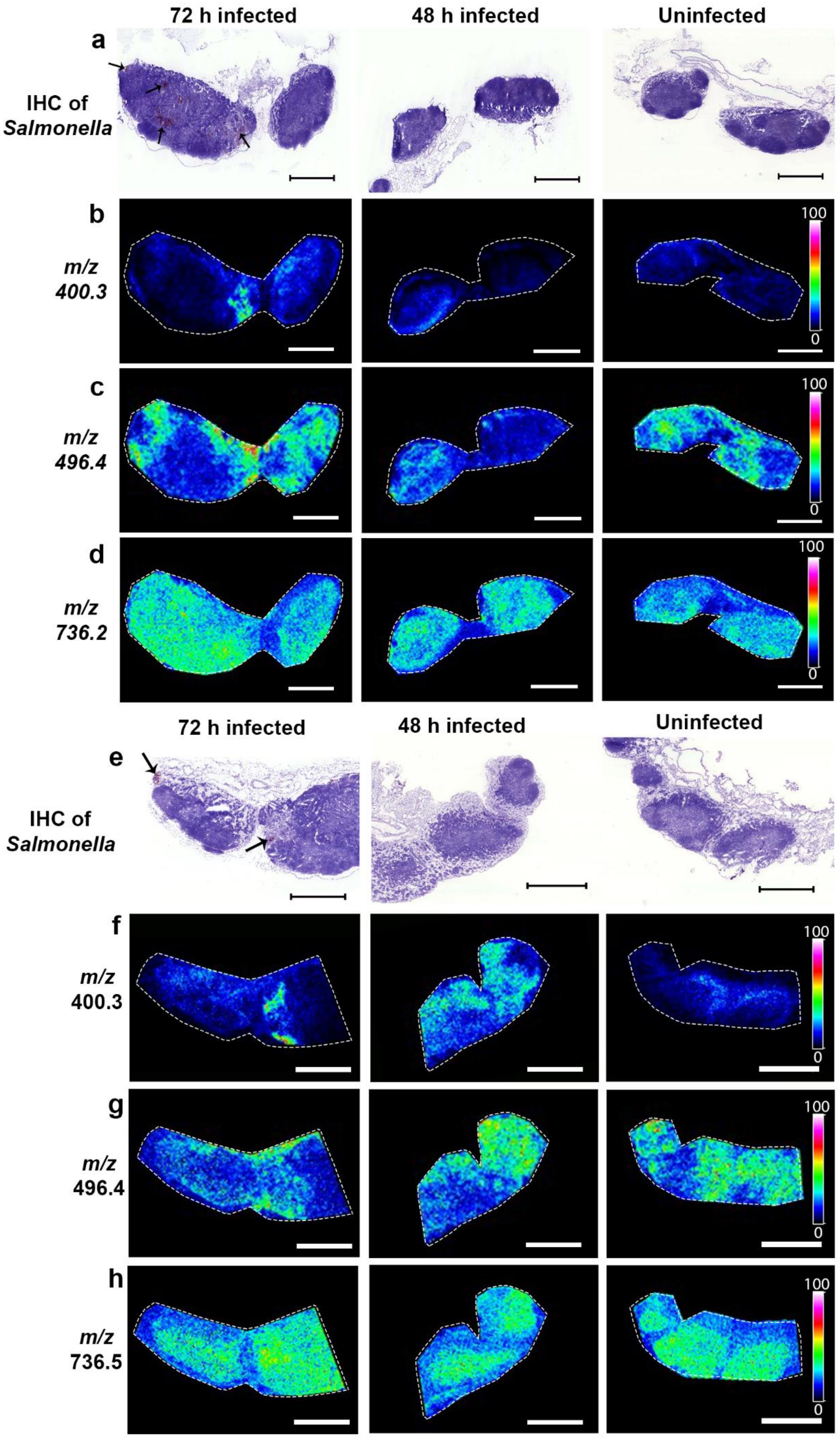
72 h infected
48 h infected
Uninfected
a
IHC of
Salmonella
b
m/z
400.3
c
m/z
496.4
d
m/z
736.2
100
0
72 h infected
48 h infected
Uninfected
e
IHC of
Salmonella
f
m/z
400.3
g
m/z
496.4
h
m/z
736.5
100
0

Supplementary Figure S1. IHC staining of *Salmonella* and MSI of the molecule of interest at *m/z* 400.3, 2 biological replicates, related to Figure 1. (**a** and **e**) IHC of *Salmonella* (red and black arrows). (**b** and **f**) MSI of molecule at m/z 400.3 in MLNs from uninfected and 48 h and 72 h *Salmonella* infected mice. MSI of control molecules at m/z 496.4 (**c** and **g**) and 736.5 (**d** and **h**) to show molecules were not ubiquitously changing in the hypocellular regions where bacteria were present. Scale bars, 1 mm.

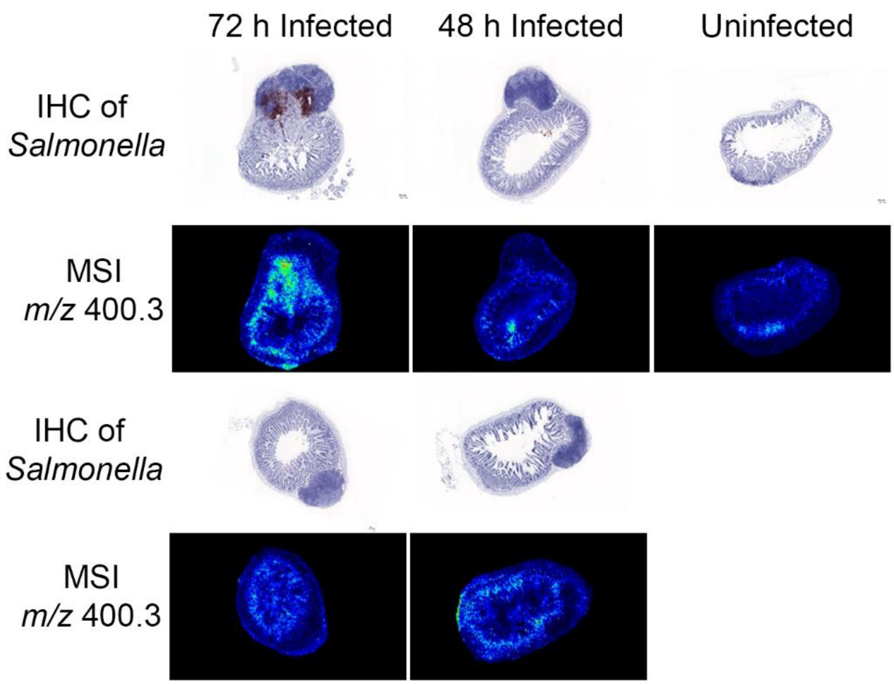

Supplementary Figure S2. IHC staining of *Salmonella* and MSI of the molecule of interest at *m/z* 400.3 in the Peyer's Patches. IHC of *Salmonella* (red) and MSI results of a molecule at *m/z* 400.3 in Peyer's patches from uninfected mice and mice infected for 48 or 72 hours with *S.* Typhimurium.

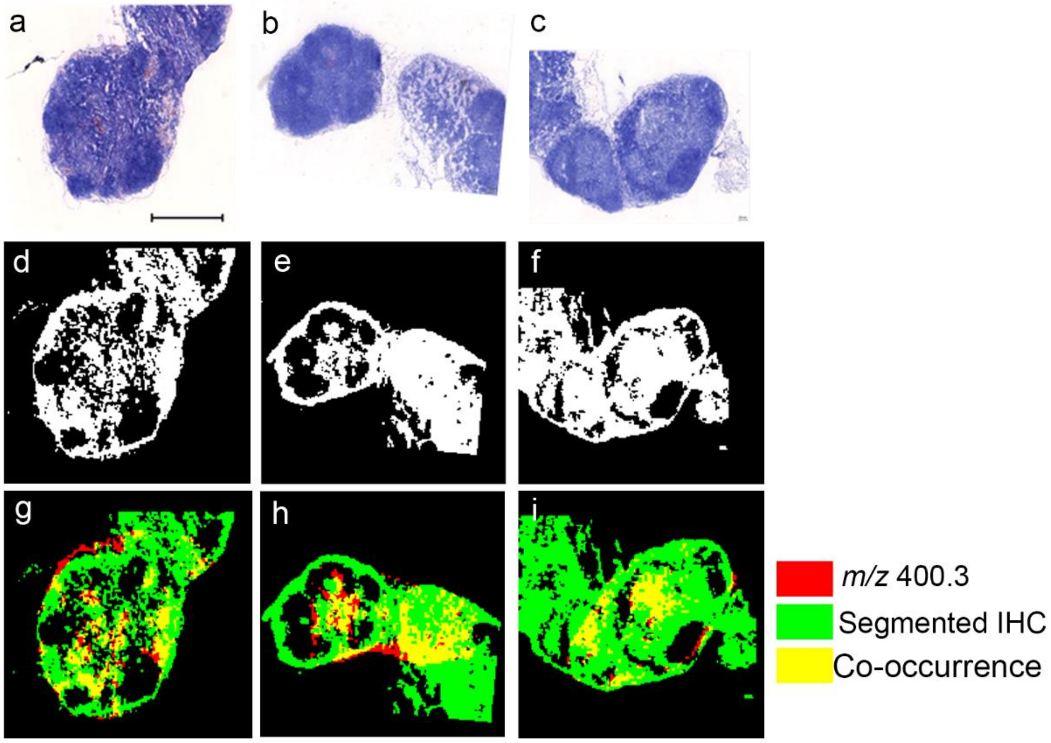

Supplementary Figure S3. Statistical analysis of co-occurrence of low cellular density regions of the lymph node with the molecule at *m/z* 400.3. (**a**-**c**) IHC images from 72 h post infection (**a**), 48 h (**b**) and uninfected (**c**). (**d**-**f**) segmented hypocellular regions from the IHC images (**a**-**c**). (**g**-**h**) degree of co-occurrence of the thresholded ion images from *m/z* 400.3 and the IHC determined hypocellular region (g 71%, h 79% and i 89%).

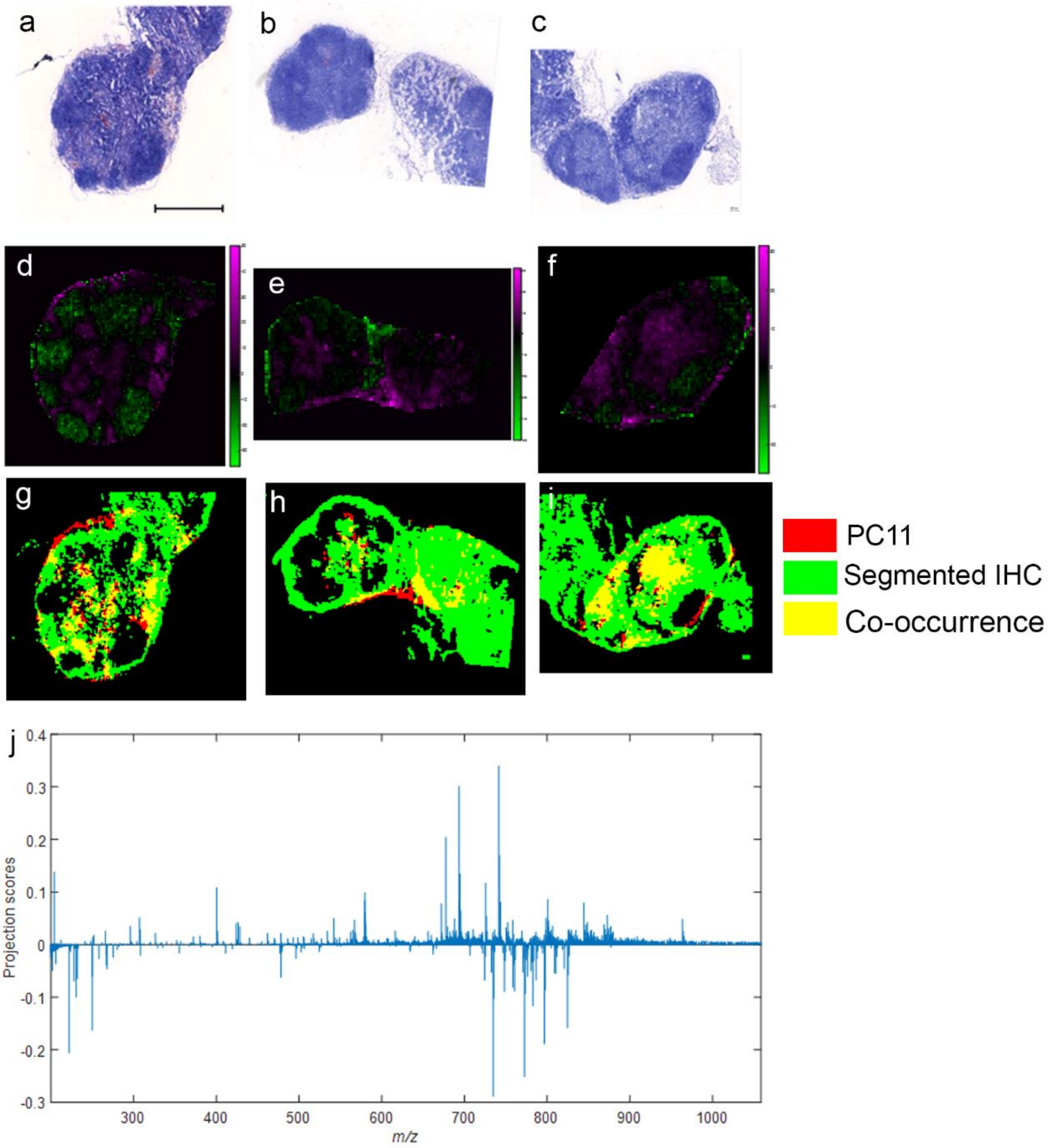

Supplementary Figure S4. Statistical analysis of co-occurrence of low cellular density regions of the lymph node with the PC11. IHC images of (**a**) 72 h infected, (**b**) 48 h infected and (**c**) uninfected MLNs. (**d**-**f**) Scored images from PC 11 of the same IHC data presented in **a**-**c**. (**g**-**h**) degree of co-occurrence of the thresholded PC 11 scores and the IHC determined hypocellular region (**g** 71%, **h** 73% and **i** 88%) (**j**) PC 11 spectral co-efficients showing a high contribution from *m/z* 400.3 along with other potential markers for hypocellularity such as *m/z* 694.55 and 725.68

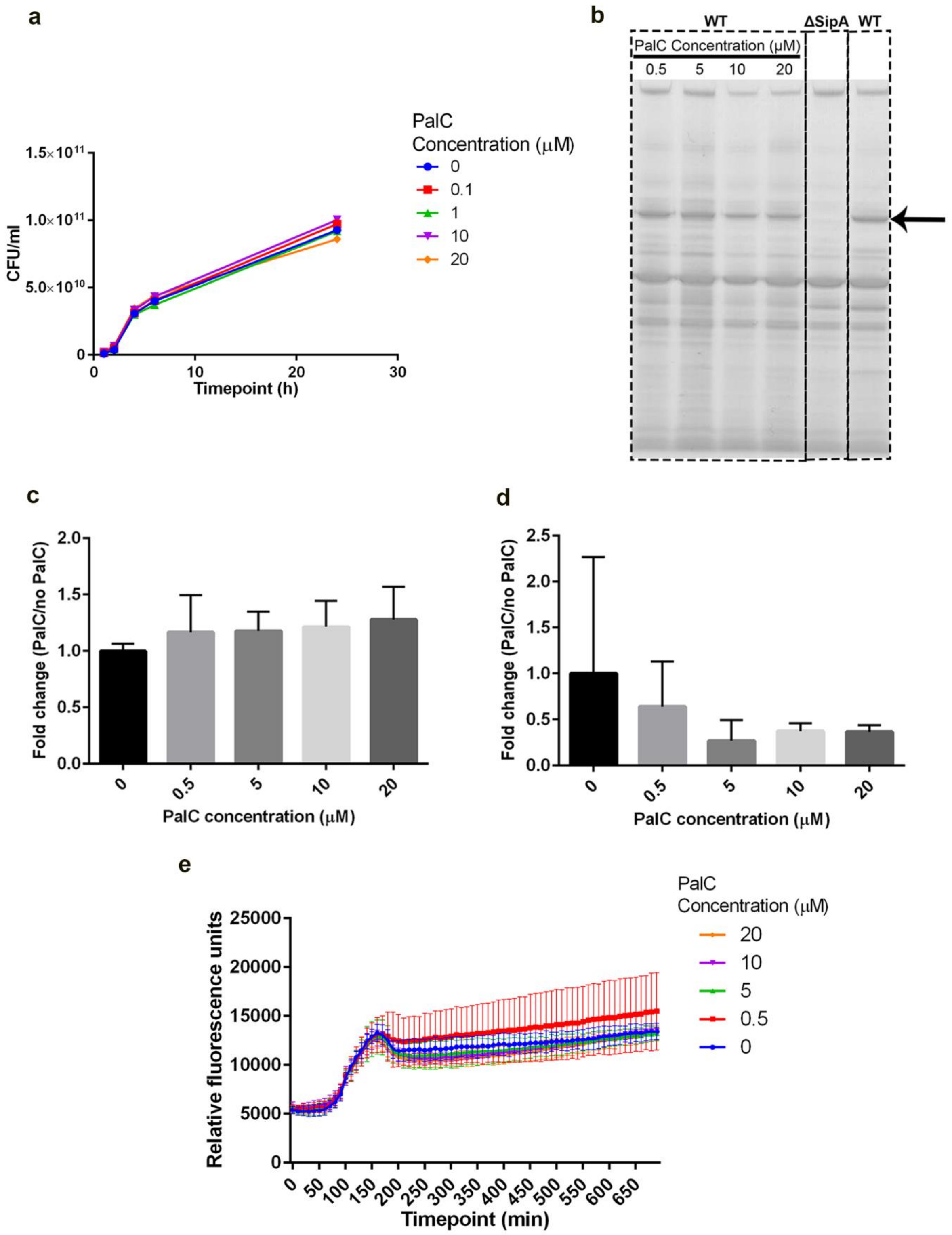

Supplementary Figure S5, The effects of PalC on Salmonella growth, virulence and motility. (**a**) Growth curve of S. Typhimurium (SL1344) in the presence of various concentrations of PalC. (**b**) SDS-PAGE analysis of levels of SipA (arrow) released from *S.* Typhimurium (SL1344) treated with various concentration of PalC. (**c**) Fold change in

swimming area of *S.* Typhimurium (SL1344), with addition of various concentration of PalC to the agar plate, 9 compared to untreated control. (**d**) Fold change in twitching area of S. Typhimurium (SL1344), with addition of various concentration of PalC to the agar plates, compared to untreated control. (**e**) Levels of FliC expression by *S*. Typhimurium (SL1344), with FliC tagged with GFP, grown in the presence of various concentrations of PalC.

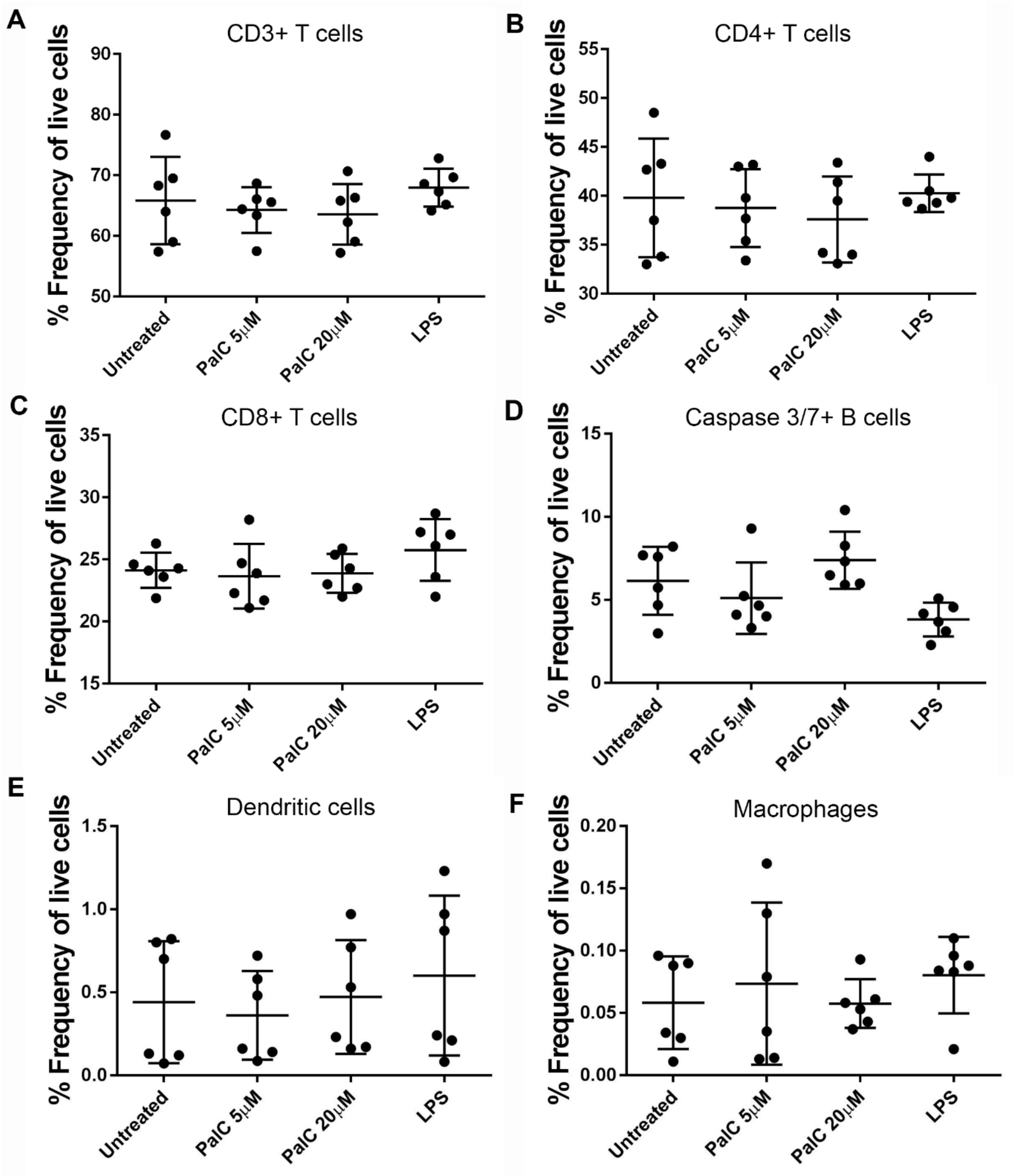

Supplementary Figure S6 PalC effects on MLN B and T cells, macrophages and DCs *ex vivo* after PalC treatment. Effect of PalC treatment on CD3$^+$ T cells **(a)**, gating: single cells> live cells> CD45$^+$ cells> CD3$^+$ cells. CD3$^+$CD4$^+$ T cells **(b)**, gating: single cells> live cells> CD45$^+$ cells> CD3$^+$ cells> CD4$^+$ cells. CD3$^+$CD8$^+$ T cells **(c)**, gating: single cells> live cells> CD45$^+$ cells> CD3$^+$ cells> CD8$^+$ cells. Caspase-3/7 levels in B220$^+$CD19$^+$ cells **(d)**, gating: single cells> live cells> CD45$^+$ cells> B220$^+$CD19$^+$ cells> caspase -3 and -7$^+$ cells. Dendritic cells **(e)**, gating: single cells> live cells> CD45+ cells> MHCII+CD11c+ cells> F480

low, B220- cells. Macrophages **(f)**, gating: single cells> live cells> CD45+cells> MHCII+CD11c- cells> F480 high, B220- cells. For all graphs N=6, 6 biological replicates from 2 separate experiments and error bars show standard deviation. Multiple comparison tests were performed for cells in all graphs but no differences were significant.

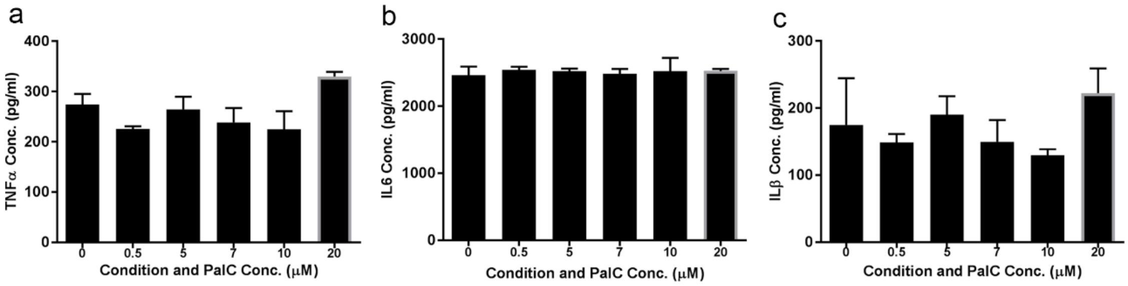

Supplementary Figure S7. The effect of PalC on inflammatory cytokine release by macrophages. The effect of treatment of RAW 264.7 macrophages with various concentrations of PalC on the release of tumour necrosis factor α (TNFα) (**a**), interleukin-6 (IL6) (**b**), and interleukin- 1β (IL1β) (**c**).

References

1. Race, A. M. *et al.* SpectralAnalysis: Software for the Masses. *Anal. Chem.* **88,** 9451–9458 (2016).

2. Beckham, K. S. H. *et al.* The metabolic enzyme AdhE controls the virulence of Escherichia coli O157:H7. *Mol. Microbiol.* **93,** 199–211 (2014).

3. Roe, A. J. *et al.* Heterogeneous Surface Expression of EspA Translocon Filaments by Escherichia coli O157:H7 Is Controlled at the Posttranscriptional Level. *Infect. Immun.* **71,** 5900–5909 (2003).
